# Not as Stable as We Think: A Descriptive Study of 12 Monthly Assessments of Fear of Cancer Recurrence Among Curatively-Treated Breast Cancer Survivors 0–5 Years After Surgery

**DOI:** 10.3389/fpsyg.2020.580979

**Published:** 2020-11-02

**Authors:** José AE Custers, Linda Kwakkenbos, Winette TA van der Graaf, Judith B Prins, Marieke FM Gielissen, Belinda Thewes

**Affiliations:** ^1^ Radboud Institute for Health Sciences, Department of Medical Psychology, Radboud University Medical Center, Nijmegen, Netherlands; ^2^ Behavioural Science Institute, Clinical Psychology, Radboud University, Nijmegen, Netherlands; ^3^ Department of Medical Oncology, Netherlands Cancer Institute – Antoni van Leeuwenhoek, Amsterdam, Netherlands; ^4^ Radboud Institute for Health Sciences, Department of Medical Oncology, Radboud University Medical Center, Nijmegen, Netherlands; ^5^ Radboud Institute for Health Sciences, Department of Primary and Community Care, Radboud University Medical Center, Nijmegen, Netherlands; ^6^ Siza (disability service) Arnhem, Arnhem, Netherlands; ^7^ Psycho-Oncology Co-operative Research Group, The University of Sydney, Sydney, NSW, Australia

**Keywords:** fear of cancer recurrence, individual trajectories, oncology, breast cancer survivors, Cancer Worry Scale, latent class growth analysis

## Abstract

**Purpose**: Previous studies suggest one-third of breast cancer survivors (BCS) experience elevated fear of cancer recurrence (FCR) and that it remains stable. Most studies include long assessment intervals and aggregated group data. This study aimed to describe the individual trajectories of FCR when assessed monthly using both a statistical and descriptive approach.

**Methods**: Participants were curatively-treated BCS 0–5 years post-surgery. Questionnaire data were collected monthly for 12 months. Primary outcome was FCR [Cancer Worry Scale (CWS)]. For the descriptive approach, 218 participants were classified as low (CWS ≤ 13 at each assessment), high (CWS ≥ 14 at each assessment), or fluctuating FCR (CWS scores above and below cut-off). Latent class growth analysis (LCGA; *n* = 377) was conducted to identify trajectories over time.

**Results**: Around 58% of the women reported fluctuating CWS scores, 22% reported a consistently high and 21% consistently low course. Results of the LCGA confirmed the three-class approach including a stable high FCR group (13%), a low FCR group (40%), and a moderate FCR group (47%). Both the moderate and low scoring groups reported declining scores over time. Younger patients, higher educated patients, and those less satisfied with the medical treatment were more likely to belong to the moderate or high trajectory.

**Conclusion**: Assessed monthly, the majority of BCS report fluctuating levels of FCR. Stepped-care models should assess FCR on multiple occasions before offering tailored interventions.

## Introduction

Fear of cancer recurrence (FCR) is one of the most prevalent symptoms among breast cancer survivors (BCS), with a high need for support (Armes et al., 2009; Boyes et al., 2012; Willems et al., 2016). While some degree of FCR is normal and adaptive, higher levels of FCR are associated with distress, psychological symptoms, rumination, excessive frequent bodily-checking, lower quality-of-life, and functional impairment (Simard et al., 2013). The reported prevalence of at least moderate FCR among Dutch BCS ranges between 31 and 56% (Van den Beuken-van Everdingen et al., 2008; Custers et al., 2014). Intuitively, one might expect FCR to reduce over time due to psychological adjustment and diminishing objective risk of breast cancer recurrence. However, a proportion of long-term BCS continue to experience FCR many years after their cancer diagnosis. For example in a large sample (*n* = 2,671) of German BCS, 17% reported moderate to high levels of fear of recurrence when surveyed an average of 8 years after diagnosis (Koch et al., 2014). While some attention has been given to studying the course and trajectories of distress (Henselmans et al., 2010; Lam et al., 2012), mental and physical functioning (Helgeson et al., 2004), and depressive symptoms (Stanton et al., 2015) among BCS, the course of FCR has received relatively little attention in the literature to date.

Synthesis of existing literature on longitudinal data of FCR in BCS is complicated by the fact that a variety of instruments have been used to assess FCR, as well as the variation in the timing and number of data collection points. Two literature reviews including studies with mixed cancer types concluded that high FCR remains stable over time (Koch et al., 2013; Simard et al., 2013). Among 22 longitudinal studies of FCR, Simard and colleagues found the majority (*n* = 18) reported either no change in FCR during the follow-up periods examined (3 months–6 years after end of treatment) or that there was an initial decrease of FCR with scores remaining stable thereafter. In a literature review by Koch et al. (2013) of FCR in long-term cancer survivors only two (Bowman et al., 2004; Langeveld et al., 2004) out of eight (Deimling et al., 2002; Bowman et al., 2004; Langeveld et al., 2004; Carver et al., 2006; Deimling et al., 2006a,b; Crespi et al., 2008; Skaali et al., 2009) longitudinal studies found that time since diagnosis was significantly associated with FCR, which the authors interpreted as evidence that FCR persists over an extensive period of time after the initial diagnosis without significant changes in intensity. Fifteen studies have examined the course of FCR exclusively among BCS with equivocal findings. In seven studies, FCR decreased over time (Bloom et al., 2004; Lebel et al., 2007; Armes et al., 2009; Lebel et al., 2009; Melchior et al., 2013; Halbach et al., 2016; Yang et al., 2018). Four studies (Stanton et al., 2002; Costanzo et al., 2007; Sheppard et al., 2009; Ashing et al., 2017) found that FCR remained stable over time, and two studies (Rabin et al., 2004; Dunn et al., 2015) found an initial decrease followed by stabilization. One study, which assessed FCR at multiple time points in the month prior to and after a follow-up mammography, found an increase then decrease in FCR after a good (i.e., negative) test result and increase during the month following the mammography (McGinty et al., 2016). Finally, one study reported stable levels of FCR in the first 6 months after surgery. After this period, older BCS reported decreasing levels of FCR whereas younger BCS reported an increase of FCR (Starreveld et al., 2018). It is noteworthy, however, that most longitudinal studies of FCR reported changes in FCR based on the mean of FCR scores or prevalence of high FCR (i.e., score above a cut-off on a questionnaire) aggregated at group level. Furthermore, most longitudinal studies have employed relatively large intervals between assessments.

Despite previous literature reviews concluding that high FCR remains stable over time, data on BCS are not consistent and equivocal on this issue. Furthermore, clinical experts often report that in their practice many patients with high FCR experience fluctuations in their level of FCR over time. Known triggers of FCR include internal cues such as fatigue, pain, fever, and other somatic symptoms, or external cues such as medical investigations, reminders of cancer, cancer in the media, and hearing about cancer diagnosis in a friend or relative (Lee-Jones et al., 1997; Custers et al., 2017).

To date, only three studies involving women with breast cancer have investigated trajectories of FCR using prospective data or used more than three follow-up assessments to describe the course of FCR. A study by Dunn et al. (2015) examined the trajectories of FCR of 396 women with breast cancer using monthly assessments of FCR in the first 6 months following breast cancer surgery. This study found that FCR scores declined significantly between the peri-operative period and 6 months after surgery, but that FCR scores plateau at approximately 4 months. Women with better physical health and those with higher FCR scores at baseline reported a steeper decline in FCR scores. Despite a significant decrease in mean levels of FCR over the first 6 months after diagnosis, the authors identified there was considerable variation in the individual trajectories of FCR scores with some women describing a highly fluctuating course.

One additional study has documented the course of FCR over the first 18 months after diagnosis in a cohort of cancer survivors. Savard and Ivers (2013) surveyed a mixed sample of 962 cancer survivors (48% breast cancer) scheduled to undergo surgery on FCR peri-operatively and again 2, 6, 10, 14, and 18 months later. FCR levels were found to be highest at baseline, and significantly decreased at the 2-month evaluation but remained stable throughout the remainder of the study for patients with both clinical and sub-clinical FCR. Patients with high FCR at baseline continued to display high levels at all subsequent time points, suggesting a chronic course among those with elevated FCR at baseline.

Finally, a recent study of Yang et al. (2018) examined FCR levels in a longitudinal design with breast cancer patients receiving radiotherapy (RT). Patients filled out a FCR questionnaire at their first day of treatment, and then weekly throughout the period of RT and 6–8 weeks after the end of treatment. Most women experienced a decline in fear during and after RT. However, there was considerable variation of trajectories observed. Initial level of FCR was the strongest predictor of follow-up FCR into the first 2 months of “survivorship” which, according to the authors, tended to support the view that FCR is quite stable and already present at the start of RT.

The results of the previous studies on the course of FCR suggest that FCR scores are highest in the peri-operative period followed by an initial decrease early in the treatment trajectory. However, despite some initial decrease, it appears that individuals with elevated FCR at baseline are likely to continue to have elevated FCR over the first 18 months post diagnosis. While together these studies provide valuable insight into the course of FCR, neither they do not identify the proportion of patients with a fluctuating course of FCR nor examine the course of FCR beyond the first 18 months after surgery.

The objective of this study was to describe the course of FCR in women with breast cancer 0–5 years post diagnosis. Specific aims were to assess:

1. Whether significant differences in mean level of FCR occur when FCR is assessed monthly for 12 months (course of FCR).
2. Whether distinct classes of individual trajectories of FCR can be identified using a statistical approach.
3. The proportion of women with a low and stable FCR (henceforth called “low FCR”), high and stable FCR (henceforth “high stable FCR”), and with levels of FCR which fluctuate above and below a validated cut-off (henceforth “fluctuating FCR”) using a more descriptive approach.

It was hypothesized that:

- Mean FCR scores would fluctuate over time when assessed at monthly intervals for 12 months.
- A higher proportion of women <3 years after surgery would have a fluctuating FCR or high FCR compared with those 3–5 years post-surgery.
- The degree of FCR fluctuation in FCR scores would be inversely associated with time since surgery. More specifically, a moderate to large negative correlation (*r* = > 0.3) between the absolute change in FCR score and time since surgery was predicted.

Since relatively little is known about how known triggers of FCR interact with the course of FCR, this study also sought to identify the self-reported triggers of FCR experienced by women with low FCR, high stable FCR, and those with fluctuating FCR.

## Materials and Methods

### Participants and Procedure

Institutional human ethics committee approval was obtained prior to commencement of the study (CMO Regio Arnhem-Nijmegen, 2012/227). Potential participants were identified by searching the institutional databases of one academic and two regional hospitals in the Netherlands for women who met the eligibility criteria for this study. Eligible participants were: 0–5 years after surgery for breast cancer; treated with curative intent and disease-free at the time of participation; and able to provide informed consent and read and write in Dutch. Eligible participants received a mailed invitation letter from their treating oncologist or surgeon explicitly stating that women both with and without elevated FCR could participate. Consenting participants received a monthly questionnaire for 12 months including demographic, medical, and psychosocial variables. Baseline questionnaires were completed in paper and pencil format, and subsequent questionnaires could either be filled in online or in paper-and-pencil form according to participants’ preference. Of the 1,205 eligible women who were invited to the study, 565 (47%) were interested in receiving more information about the study and 460 (38%) consented. Study participants were compared to 539 non-responders (data of one regional hospital were not available) demonstrating that participants were significantly [*t*(993,635) = 5.77, *p* < 0.001] younger (*M* = 56.69, *SD* = 9.6) than non-responders (*M* = 60.64; *SD* = 11.9).

### Sample Sizes

To be included in the statistical approach of class distinction (LCGA) which can handle missing data correctly, at least seven completed assessments and no more than two consecutive missing assessments were required. Therewith, longitudinal data of 377 patients were analyzed.

For the descriptive analysis in which it was not desirable to have missing data, patients with incomplete data for the Cancer Worry Scale (CWS) on any of the 12 time points were excluded resulting in a sample of 218 participants.

### Measures

#### Clinical and Demographic Variables

Patients completed socio-demographic items assessing age, marital status, having children, education, and employment status as part of the baseline questionnaire. Clinical variables, including type of treatment and time since surgery, were self-reported in the baseline questionnaire.

#### Fear of Cancer Recurrence

Fear of cancer recurrence severity was evaluated using the CWS. The CWS is used in research to assess concerns about developing cancer again and the impact of those concerns on daily functioning (Lerman et al., 1991; Douma et al., 2010). The eight items of the CWS are rated on a four-point Likert scale ranging from “*never*” to “*almost always*.” Scores range from 8 to 32. The CWS has been validated in Dutch women with breast cancer (Custers et al., 2014). A cut-off score of 14 or higher (sensitivity 77%; specificity 81%) has been validated in women with breast cancer indicating a high level of FCR and applied in this study (Custers et al., 2014). In this study, the Cronbach’s alpha varied between 0.86 and 0.89.

#### Descriptive Classification of FCR Course

The course of FCR for each participant was classified using the following purpose-designed *a priori* criteria:

- Low stable: CWS score of 13 or lower at each assessment during the 12 month assessment period.
- High stable: CWS score of 14 or above at each assessment during the 12 month assessment period.
- Fluctuating: CWS scores both above and below cut-off for high FCR at subsequent assessments during the 12 month assessment period.

#### Triggers of FCR

The 12 month questionnaire asked participants to complete a short open-ended question “*Has there been a particular situation or event that may have influenced the degree of fear that you experience now*?” immediately after completing the CWS.

### Data Analysis

#### Absolute Change in FCR Scores (Total Delta)

To calculate the total magnitude of change in FCR scores over time, an absolute change score (delta) was calculated for each interval between months 1 to 12 in the data collection. A total absolute change score (total delta) was calculated by summation of the 11 monthly delta scores between months 1 to 12.

#### Identification of Classes

Following the guidelines described by Jung and Wickrama (2008), latent class growth analysis (LCGA) was conducted using MPlus to identify trajectories (classes) over time for CWS scores. MPlus’ full information maximum likelihood estimation for handling missing data was applied. By estimating individual variability in outcome over time, individuals are classified into latent classes based upon similar patterns of FCR.

A single-class growth curve model, as well as a three-class model was specified. To determine the number of classes in the sample, the three-class model was compared with a two-class and four-class model, as well as a five- and six-class model. In total, the fit of six unconditional latent class models (i.e., models with no covariates) was estimated, with one to six linear trajectories. The number of trajectories was determined based on model parsimony, fit indices, and clinical interpretability. The best fitting model has significant *p*-values (*p* < 0.05) for the Bootstrap Likelihood Ratio Test (BLRT) and the Vuong-Lo-Mendell Ruben Likelihood Ratio Test (LMR-LRT), the smallest Bayesian Information Criterion (BIC), a higher entropy statistic (near 1.0), and higher posterior probabilities of group membership (near 1.0). The number of participants (not less than 5% of total sample) of the identified classes was considered for clinical interpretability.

#### Baseline Characteristics of Classes

Based on literature (Simard et al., 2013), we compared *a priori* defined baseline demographic and medical characteristics between the identified classes using univariate and multivariate logistic regression analyses. First, univariate associations of baseline characteristics (age, partnered, children, education, employment, time since diagnosis, additional treatment, and satisfaction with medical treatment) with the classes of FCR were calculated. Next, to understand the independent contribution of the baseline characteristics, multivariate logistic regression analyses were conducted.

Latent class growth analysis was performed in Mplus 7 and the logistic regression analyses in SPSS version 25.

#### Classification of Low, Fluctuating, or High Stable FCR: Descriptive Approach

Chi-square was used to examine difference in the proportions of women who were classified as having low and stable, fluctuating, or chronically elevated FCR by time since surgery (<3 vs. 3–5 years). Associations between absolute change in FCR (total delta) and time since diagnosis were explored using Pearson’s correlation. Differences in mean delta scores between women with a low, fluctuating, and high level of FCR were assessed with one way between groups ANOVA with *post hoc* contrasts.

#### Triggers of FCR

Reported triggers were independently coded for themes by two researchers. Coding was initially conducted using *a priori* codes derived from the triggers subscale of the FCR Inventory (FCRI; Simard and Savard, 2009). Initial coding was discussed by the research team, and where necessary codes were adapted or new codes added, following which both raters re-coded all responses and ratings were compared to check for inter-rater agreement (87%). Any further discrepancies were resolved through discussion until consensus was achieved.

## Results

### Participant Characteristics

Longitudinal data of 377 patients were available for the statistical approach of class distinction. Demographics and medical characteristics of these participants are shown in Table 1.

**Table 1:** Baseline characteristics of the identified subgroups of fear of cancer recurrence (FCR).

	Total *n* = 377	Low declining FCR *n* = 149	Moderate declining FCR *n* = 177	High stable FCR *n* = 51	
Demographics					
Age, mean (SD), years^*^	57.6 (9.4)	58.8 (9.2)	56.2 (9.0)	58.9 (10.3)	*F*(2,373) = 3,83, *p* = 0.02
Married/partnership, *n* (%)	280 (75%)	110 (74%)	129 (74%)	41 (80%)	*Χ* ^2^(2) = 0.89, *p* = 0.64
Children, *n* (%)^*^	313 (84%)	116 (78%)	150 (86%)	47 (92%)	*Χ* ^2^(2) = 6.26, *p* = 0.04
Education level^*^					
High, *n* (%)	110 (30%)	55 (38%)	44 (25%)	11 (22%)	*Χ* ^2^(2) = 10.86, *p* = 0.03
Currently employed, *n* (%)	174 (46%)	68 (45%)	88 (50%)	18 (35%)	*Χ* ^2^(2) = 3.46, *p* = 0.18
Medical characteristics					
Time since diagnosis, mean (SD), years	2.8 (1.3)	2.8 (1.3)	2.8 (1.3)	2.6 (1.3)	*F*(2,365) = 0.492, *p* = 0.61
Additional treatment^a^					
Chemotherapy, *n* (%)	267 (71%)	107 (73%)	124 (71%)	36 (71%)	*Χ* ^2^(2) = 0.23, *p* = 0.89
Radiotherapy, *n* (%)	289 (78%)	115 (78%)	136 (78%)	38 (75%)	*Χ* ^2^(2) = 0.31, *p* = 0.86
Hormonal therapy, *n* (%)	237 (63%)	90 (61%)	119 (68%)	28 (55%)	*Χ* ^2^(2) = 3.23, *p* = 0.19
Trastuzumab, *n* (%)	45 (12%)	18 (12%)	19 (11%)	8 (16%)	*Χ* ^2^(2) = 0.91, *p* = 0.64
Psychosocial factors					
Satisfaction with medical treatment (0 not at all–4 very satisfied)^*^	3.4 (0.7)	3.5 (0.7)	3.3 (0.7)	3.2 (0.9)	*F*(2,370) = 4,79, *p* = 0.01

a Does not sum to 100% as respondents could endorse multiple categories.

* *p* < 0.05.

For the descriptive analysis, a total of 218 women with complete CWS data were analyzed. Characteristics of the *n* = 377 sample were comparable to the participants for the descriptive analysis (*n* = 218): mean age was 58 years on average at baseline (range 33–87 years, *SD* = 9.4) and patients were on average 2.8 years post-diagnosis (range 0.5–5.9 years, *SD* = 1.4). The majority of participants was married or partnered (77%) and had children (82%). Approximately half (49%) had moderate education; one quarter had low (25%) and approximately one quarter (26%) higher education. Participants received a variety of adjuvant therapies including chemotherapy (65%), radiotherapy (78%), hormonal therapy (63%), and trastuzumab (10%).

### Course of FCR

For the complete sample (*N* = 377), the intercept of the CWS score generated with Mplus was 14.1 (95% CI 13.7–14.5), indicating moderate to high FCR. There was a slight decrease in CWS score (less FCR) over time (slope −0.07; 95% CI −0.09 to −0.04), equivalent to an average decrease in CWS score of −0.84 per year.

### Identification of Classes: Statistical Approach

A three-class model was identified as most appropriate based on fit indices, internal reliability, and interpretability (Table 2). In the three-class model, compared with the two-class model, the BIC was better, but the entropy was lower. The posterior probabilities were similar for the two and three-class model. Compared with the four-class model, in the three-class model, the BIC was somewhat lower, whereas other fit indices were highly similar. In the four-class model, however, a subgroup was identified including only 10 patients, limiting clinical interpretability.

**Table 2:** Fit indices, entropy, and average posterior probabilities across models with different number of classes with distinct trajectories of cancer worry.

No. of classes	BIC	LMR-LRT	BLRT	Entropy	*n*	Posterior probabilities	Intercept (95% CI)	Slope linear (95% CI)
2	21117.46	0.002	<0.0001	0.968	102 (27.1%) 275 (72.9%)	0.98 1.00	18.6 (17.8, 19.5) 12.5 (12.2, 12.8)	−0.07 (−0.13, −0.004) −0.08 (−0.10, −0.05)
3	19868.72	0.010	<0.0001	0.950	177 (47.0%) 51 (13.5%) 149 (39.5%)	0.98 0.99 0.97	14.9 (14.5, 15.4) 20.4 (19.6, 21.3) 10.9 (10.5, 11.3)	−0.09 (−0.12, −0.05) −0.03 (−0.13, 0.06) −0.06 (−0.09, −0.03)
4	19348.81	0.014	<0.0001	0.955	61 (16.2%) 135 (35.8%) 171 (45.4%) 10 (2.7%)	0.98 0.97 0.98 1.00	18.6 (17.7, 19.5) 10.8 (10.3, 11.2) 14.5 (14.1, 15.0) 24.1 (22.8, 25.5)	−0.04 (−0.13, 0.06) −0.07 (−0.10, −0.03) −0.09 (−0.13, −0.06) 0.05 (−0.13, 0.24)
5	18984.81	0.012	<0.0001	0.935	81 (21.5%) 41 (10.9%) 140 (37.1%) 105 (27.9%) 10 (2.7%)	0.95 0.98 0.94 0.97 1.00	15.9 (15.2, 16.7) 19.5 (18.9, 20.1) 13.5 (12.9, 14.1) 10.3 (10.0, 10.7) 24.1 (22.8, 25.5)	−0.06 (−0.14, 0.02) −0.05 (−0.16, 0.06) −0.10 (−0.14, −0.06) −0.06 (−0.10, −0.03) 0.06 (−0.13, 0.24)

BIC, bayesian information criterion; LMR-LRT, vuong-lo-mendell rubin likelihood ratio test; BLRT, bootstrap likelihood ratio test; and CI, confidence interval.

The three subgroups differed in the baseline values (intercepts) of the CWS scores. The first subgroup consisted of 149 participants and was defined as “low declining FCR,” as participants reported low baseline CWS scores (intercept 10.9; 95% CI 10.5–11.3), and the slope was −0.06 (95% CI −0.09 to −0.03). The second subgroup was defined as “moderate declining FCR,” as the 177 participants in this group showed moderate to high baseline CWS scores (intercept 14.9; 95% CI 14.5–15.4), and the slope was −0.09 (95% CI −0.12 to −0.05). The third subgroup was defined as “high stable FCR.” For this subgroup of 51 patients, the intercept was 20.4 (95% CI 19.6–21.3), and the slope was non-significant (−0.03; 95% CI −0.13 to 0.06).

### Baseline Characteristics of Classes

The results of the univariate and multivariate regression analyses comparing baseline characteristics of participants between the three FCR classes are shown in Table 3. Four variables (age, education, children, and satisfaction with medical treatment) were significantly different between groups in the univariate analysis and therefore selected to be included in the final model with the low declining trajectory as the reference group. The final model was statistically significant (*χ* ^2^ = 27.934, *df* = 8, *p* < 0.001, Cox and Snell *R* ^2^ = 0.074, Nagelkerke = 0.086, and McFadden = 0.039). Age, education, and satisfaction with medical treatment remained significant predictors of FCR trajectory. Younger patients (moderate declining OR = 0.967; high stable OR = 0.994), higher educated patients (moderate declining OR = 1.954; high stable OR = 2.083), and patients less satisfied with medical treatment (moderate declining OR = 0.656; high stable OR = 0.579) were more likely to belong to the moderate declining or stable high trajectory than the low declining trajectory.

**Table 3:** Final model of baseline characteristics associated with subgroup membership for FCR, multinominal regression analysis for FCR (moderate declining worry and high stable worry vs. low declining worry).

	*Χ* ^2^	*p*	*B*	Wald	Exp (*B*)	95% CI
Age	7.591	0.022				
Moderate declining			−0.033	6.831	0.967	0.943–0.992
High stable			−0.006	0.111	0.994	0.959–1.030
Children	4.044	0.132				
Moderate declining			−0.432	1.898	0.650	0.352–1.200
High stable			−0.962	2.860	0.382	0.125–1.165
Education	8.161	0.017				
Moderate declining			0.670	6.947	1.954	1.187–3.215
High stable			0.734	3.538	2.083	0.970–4.475
Satisfaction medical treatment	7.603	0.022				
Moderate declining			−0.421	5.339	0.656	0.459–0.938
High stable			−0.546	5.379	0.579	0.365–0.919

### Descriptive Course of FCR

Of the 218 women with CWS scores on all 12 assessments, approximately one-fifth of the sample (*n* = 45, 20.6%) reported low FCR at each monthly assessment (CWS scores 13 or lower). A similar proportion (*n* = 47, 21.6%) consistently scored above cut-off on the CWS (high FCR) at each time point, while the majority (*n* = 126, 57.8%) reported scores which fluctuated above and below the validated CWS cut-off score for high FCR over 12 monthly assessments. No significant differences were observed in the proportion of women classified as low, fluctuating, or high CWS scores between those who were <3 years since surgery and women who were 3–5 years post-surgery (*χ* ^2^ = 1.68, *p* = 0.43).

Mean CWS scores for the entire sample ranged from 13.2 to 14.8 across the assessment period (12 months).

### Absolute Change in FCR Scores Over 12 Months

The median absolute change in CWS over 12 months (total delta) was 16 CWS points (*M* = 17.5, SD = 9.49, range = 0–67). There was no association between total delta and time since diagnosis. A one-way between-groups ANOVA was conducted to assess differences in mean delta scores between women with a low, fluctuating, and high level of FCR. There was a statistically significant difference between the groups: *F*(2, 215) = 23.96, *p* < 0.001. *Post hoc* comparisons indicated that the mean delta scores for the stable high (*M* = 21.0, *SD* = 8.5) and fluctuating group (*M* = 18.9, *SD* = 9.6) were significantly higher compared to the stable low group (*M* = 9.7, *SD* = 4.9).

### Triggers of FCR

Fifty women (23%) reported an identifiable trigger at the final assessment. Trigger themes emerging from the data were: (1) medical appointments or examinations; (2) change of medication; (3) conversations about cancer or illness; (4) seeing or hearing about someone who is ill; (5) feeling unwell (physical symptoms); (6) funerals obituaries; or (7) other. The most commonly reported triggers were seeing or hearing about someone else who is unwell (reported 16 times), hearing of a death (12 times), or personally feeling unwell or experiencing physical symptoms (8 times). Women classified as having below cut-off CWS score (low FCR) at each assessment reported fewer triggers than those with high stable or fluctuating FCR, and many categories of triggers endorsed by women with high FCR or fluctuating FCR were not endorsed at all by those classified as having low FCR (e.g., physical symptoms, funerals, or obituaries).

## Discussion

The main finding of this study was that more than half (58%) of BCS in the present study reported CWS scores which fluctuated above and below a validated cut-off for high FCR at each monthly assessment over 12 months, while approximately one-fifth reported high scores and one-fifth reported low scores at all time points. These findings are partly in line with those of Savard and Ivers (2013) who found among a mixed cancer survivors group that patients with clinical FCR at baseline continued to display clinical levels at all subsequent time points.

Contrary to our hypothesis that fluctuation in CWS scores would decrease over time as women were expected to adjust to their breast cancer diagnosis, we found no significant association between absolute change in CWS scores over 12 months and time since diagnosis. Nor was a significant difference observed in the proportion reporting high, low, and fluctuating course up to 3 years post-surgery compared with 3–5 years post-surgery, suggesting that fluctuation in FCR continues through the first 5 years after diagnosis. Therefore, compared to previous literature, the results of the present study suggest that high FCR may not be as stable as it has been previously characterized. Similar to the findings of Dunn et al. (2015), our data indicated that individual fluctuation of FCR is common. Descriptive analysis of absolute change in CWS scores over 12 months and plots of individual FCR scores over time suggested that the FCR scores of those with low FCR remain relatively stable. Greater variability in FCR scores was observed in women who scored above cut-off at each monthly assessment (high and stable) and those whose scores fluctuated above and below cut-off at each monthly assessment (fluctuating). This level of variability in FCR scores seemed to be characterized by the way survivors respond to triggers of FCR as triggers were more commonly reported by women who experienced high FCR at all time points and those with fluctuating levels of FCR. This could be explained by the fact that high FCR is characterized by high levels of preoccupation and being less able to respond to triggers in an adaptive way. Although, the fluctuating group also pays attention to triggers they seem better able to adapt to and normalize accompanying feelings of FCR over time. Women with low FCR at all time points spontaneously reported fewer triggers of FCR, possibly due to less exposure to triggers, paying less attention to triggers or finding them less bothersome.

Regarding the nature of triggers, in accordance with the theoretical model of Lee-Jones et al. (1997) and Custers et al. (2017), both external (seeing or hearing about someone else who is unwell) and internal triggers (personally feeling unwell or experiencing physical symptoms) were reported. This nature of triggers might also be related to the culture in the Netherlands with a lot of media attention on (breast) cancer (e.g., breast cancer month), regular medical check-ups, national screening programs for breast cancer, and most people speaking openly about cancer.

Strength of this data was that it included assessment of triggers for current rating of FCR rather than retrospective recall of triggers as has been used in most previous studies. However, a potential limitation was that women who use avoidance-based strategies for managing FCR may also have avoided participating and or answering these questions given they were optional and less than one quarter of the sample could identify specific triggers. Research concerning triggers of FCR is currently very limited but it is of high relevance to developing evidence-based theoretical models of FCR (Fardell et al., 2016; Custers et al., 2017; Simonelli et al., 2017) and improving our understanding of the evolution of FCR.

Results of the LCGA confirmed the three-class approach including a stable high FCR group (13%), a low group (40%), and a moderate group (47%). Both the moderate and low scoring groups reported declining scores over time. The moderate group might be interpreted as fluctuating with a moderate-to-high intercept of 14.9 and a slope of −0.009 resulting in a decrease of 0.11 per year, continuing around the cut-off score. Compared with patients in the low declining FCR group, younger patients, higher educated patients, and those less satisfied with the medical treatment were more likely to belong to the moderate declining or high stable trajectory. These predictors are in line with the review of Simard et al. (2013) showing moderate to strong evidence for poor healthcare satisfaction and younger age as predictors for FCR. Findings on education as predictor for FCR remained inconclusive.

### Strengths and Limitations

One of the strengths of this study is its use of a statistical (bottom-up) as well as descriptive (top-down) approach to correctly identify trajectories of FCR. Both approaches revealed three distinct trajectories of FCR, enhancing its validity and clinical interpretability. However, by interpreting the results, it is important to keep in mind that this was not an inception cohort, only linear trajectories were assessed and although the descriptive analysis was based on a validated cut-off score for high FCR, we acknowledge that the selected cut-off has not been validated against a gold-standard interview for clinical FCR. Furthermore, since participants were aware of the fact that the purpose of the study was FCR, it is plausible that there was a selection in signups for the study as confirmed by the moderate response rate of 38%. Selection bias is an aspect that should be taken into account when designing research on FCR since a proportion of survivors recognizes their FCR and expresses a need for help; whereas, other survivors cope with FCR by avoiding threat, including study questionnaires.

### Implications and Future Research

Current interventions for FCR are mostly offered on the basis of a score above cut-off for high FCR on a single screening occasion (Butow et al., 2013; Van de Wal et al., 2017). An important clinical implication of the present findings is that FCR should be assessed on multiple occasions before a healthcare professional decides that new evidence-based clinical interventions for FCR are warranted. A stepped-care model with a first stage of intervention, which may include watchful waiting, psycho-education, online interventions, or other self-management approaches might be appropriate. If FCR does not dissipate after an initial waiting period or period of less intensive intervention, more intensive face-to-face interventions could be offered. Such a model has been effectively used in a hospital setting (Krebber et al., 2012, 2016) and may produce cost-savings for the health system, and help ensure that limited resources are directed to those most in need. The results of the present study also have relevance for emerging trials of FCR interventions, and raise the question whether the results observed might simply reflect the natural fluctuations in FCR over relatively short intervals (3–4 months).

There is growing interest in the use of novel research methods using very frequent assessment of symptoms (e.g., ecological momentary assessment). ESM is a method in which participants are asked to rate their situations, emotions, and reactions at random moments during the day for multiple days (Csikszentmihalyi and Larson, 2014). Compared with retrospective questionnaires which often assess constructs “over the past week,” ESM offers several advantages: enhanced ecological validity because participants are assessed in their normal daily environment, minimized retrospective bias because participants’ experiences are assessed in the moment, and enhanced reliability because participants’ are assessed repeatedly. Assessment of FCR using ESM might be a valuable approach for future research since frequent reassessment of FCR will provide further insights into the evolution of FCR and may prove useful in future trials evaluating FCR interventions. For researchers, the results of the present study suggest that future longitudinal studies should consider assessing FCR on multiple (>2) time-points and to consider shorter durations between assessments in order to capture potential variability. A better understanding of the evolution of FCR will help guide the implementation of evidence-based treatments for FCR.

## Data Availability Statement

Data are available upon reasonable request. Requests to access the datasets should be directed to jose.custers@radboudumc.nl.

## Ethics Statement

The studies involving human participants were reviewed and approved by Commissie Mensgebonden Onderzoek Regio Arnhem-Nijmegen. The patients/participants provided their written informed consent to participate in this study.

## Author Contributions

All authors contributed to the study conception and design. Material preparation, data collection and analysis were performed by JC, MG, LK, WG, and JP. The first draft of the manuscript was written by JC, BT, and LK and all authors commented on previous versions of the manuscript. All authors contributed to the article and approved the submitted version.

### Conflict of Interest

The authors declare that the research was conducted in the absence of any commercial or financial relationships that could be construed as a potential conflict of interest.
